# Structural differences in the cortex of individuals who experience the autonomous sensory meridian response

**DOI:** 10.1002/brb3.2894

**Published:** 2023-01-24

**Authors:** Jennifer Kornelsen, Beverley K. Fredborg, Stephen D. Smith

**Affiliations:** ^1^ Department of Radiology University of Manitoba Winnipeg Manitoba Canada; ^2^ Department of Physiology and Pathophysiology University of Manitoba Winnipeg Manitoba Canada; ^3^ Department of Psychology University of Winnipeg Winnipeg Manitoba Canada; ^4^ Department of Psychology Toronto Metropolitan University Toronto Ontario Canada

**Keywords:** autonomous sensory meridian response, cortical complexity, cortical thickness, supramarginal gyrus, sensorimotor

## Abstract

**Background and purpose:**

The autonomous sensory meridian response (ASMR) is a multimodal perceptual phenomenon in which specific sensory triggers evoke tingling sensations on the scalp, neck, and shoulders; these sensations are accompanied by a positive and calming affective state. Previous functional neuroimaging research has shown that ASMR experiences involve medial prefrontal and sensorimotor brain areas. The purpose of the current study was to examine whether there are structural differences in the cortex of individuals who experience ASMR.

**Methods:**

Seventeen individuals with ASMR and 17 matched control participants completed an MPRAGE structural MRI scan. These data were analyzed to determine if group differences were present for measures of cortical thickness, cortical complexity, sulcal depth, and gyrification.

**Results:**

ASMR was associated with reduced cortical thickness in a number of regions including the left precuneus, precentral gyrus, and insula, and the right orbitofrontal cortex, superior frontal cortex, and paracentral lobule. Reduced thickness was observed bilaterally in the supramarginal gyrus. Individuals with ASMR also showed less cortical complexity in the pars opercularis and pars triangularis.

**Conclusions:**

The differences in cortical thickness and complexity were in brain areas whose functions relate to the ASMR experience. These differences include neural regions related to phonological processing, sensorimotor functions, and attention.

## Graphical abstract

The autonomous sensory meridian response (ASMR) is a perceptual phenomenon in which specific sensory triggers (e.g., whispering) evoke tingling sensations on the scalp, neck, and shoulders; these sensations are accompanied by a positive and calming affective state. In the current study, ASMR was associated with reduced cortical thickness in a number of regions related to sensation, movement, phonological processing, and emotional perception.

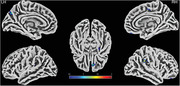

## 1 INTRODUCTION

Individuals who experience the autonomous sensory meridian response (ASMR) report that specific stimuli, known as ASMR triggers, elicit tingling sensations on the scalp, neck, and shoulders (Barratt & Davis, 2015). These sensations frequently radiate down the spine and the limbs. These sensorimotor sensations are also accompanied by a pleasant, “flow‐like” emotional state (Barratt & Davis, 2015). What sets ASMR apart from other atypical multimodal experiences is its triggers; ASMR is typically evoked by soft, low‐frequency sounds (e.g., whispering), repetitive noises, or viewing socially intimate interactions (e.g., personal grooming).

The subjective reports of ASMR experiences have been corroborated by research examining its neural substrates. Psychophysiology researchers reported that ASMR involves an increase in skin conductance responses and a reduced heart rate (Engelbregt et al., 2022, Poerio et al., 2018). Functional MRI studies have demonstrated that ASMR videos elicit activity in the anterior cingulate gyrus and sensorimotor regions including the paracentral lobule, supplementary motor area, and spinal cord (Lochte et al., 2018, Smith et al., 2019, Smith et al., 2022). Finally, research using electroencephalography has indicated that ASMR is associated with increased alpha wave activity in medial frontal regions and increased gamma wave activity in sensorimotor cortex (Smith et al., 2022), as well as with increased beta wave activity in the left temporal lobe (Engelbregt et al., 2022); decreased theta wave activity was also observed in left frontal and right temporal regions (Engelbregt et al., 2022). Together, these early investigations indicate that the experience of ASMR produces verifiable changes in neural activity in several regions of the nervous system.

Although these task‐based neuroimaging studies have helped identify the brain areas involved in the ASMR response itself, less is known about why some individuals experience ASMR “tingles” while others do not. Resting‐state functional MRI studies have demonstrated that individuals with ASMR have a greater “blending” of resting‐state networks than people who do not experience ASMR (Fredborg et al., 2021). However, these studies did not examine whether there are structural differences between individuals who experience ASMR and those who do not. The goal of the current research is to measure cortical thickness and complexity, sulcal depth, and gyrification to determine whether ASMR is associated with unique neural morphology, and to examine whether any detected differences are in brain regions identified in earlier studies as being relevant to the phenomenology of the ASMR experience (Lochte et al., 2018, Smith et al., 2019, Smith et al., 2022).

Reduced cortical thickness and complexity are typically associated with less efficient neural processing. Indeed, these cortical surface measures can reflect neuroplastic changes associated with aging (Frangou et al., 2019, Lin et al., 2021), cognition (Gautam et al., 2015), intellect and education (Im et al., 2006), and neuropsychiatric conditions (Ha et al., 2005). Importantly, differences in cortical surface measures have been shown in individuals who experience atypical conscious states similar to ASMR, with both mindfulness meditators (Kang et al., 2013) and synesthetes (Rouw et al., 2011) showing increases in cortical thickness compared with matched controls. If ASMR is associated with meditation‐like benefits, we would expect to see increased cortical thickness in frontal regions. In contrast, if ASMR is associated with less efficient control of attention—as was speculated previously (Smith et al., 2019b, Smith et al., 2017)—then reduced cortical thickness would be present in the lateral prefrontal cortex and parietal regions. Impaired attentional control may also be related to reduced sulcal depth (Voorhies et al., 2021); however, because less is known about the cognitive correlates of gyrification and sulcal depths, these analyses were exploratory in nature.

## 2 METHODS

### 2.1 Participants

Seventeen individuals who experience ASMR (nine females, eight males, *M*_age_ = 22.71; SD_age_ = 4.74) and 17 control participants (nine females, eight males; *M*_age_ = 22.76; SD_age_ = 5.39) took part in this experiment. Participants were matched for age (plus or minus three years) and biological sex. This sample size was based on effect size estimates obtained in a meta‐analysis of grey matter changes in contrasts of meditators and controls (Cohen's *d* = ∼.45 to ∼1.5; Pernet et al., 2021) and publication recommendations for neuroimaging research, at alpha 0.05 and power 0.8 (Hayasaka et al., 2007). None of the participants reported any history of neurological or psychiatric illnesses.

ASMR sensitivity was confirmed in two ways. Prior to the scanning session, each participant viewed two ASMR‐eliciting videos in the presence of one of the investigators in order to confirm that they were, or were not, ASMR sensitive. The videos consisted of a scalp check simulation (https://www.youtube.com/watch?v=P1VOe88QyP4) and a woman whispering and playing with someone else's hair (https://www.youtube.com/watch?v=yA2HcNRTdFY&t=3s). Although no physiological measurement was available during this prescreening, each ASMR‐sensitive individual was able to provide verbal descriptions of their ASMR after watching the video. Additionally, all ASMR participants showed unique patterns of neural activity in response to trimmed (4‐min) versions of these videos during later functional MRI scans (Smith et al., 2019), thereby corroborating their classification as ASMR sensitive. Participants who experienced ASMR indicated their different ASMR “triggers” using a 16‐item ASMR Checklist used in our previous investigations of the phenomenon (Fredborg et al., 2017).

Participants provided informed, written consent and completed MRI safety screening prior to entering the MRI scanner. Ethics approval was received by both the University of Manitoba and the University of Winnipeg Human Research Ethics Committees. Participants received $50 CAD (≅ $40 USD) remuneration.

### 2.2 MRI data acquisition

Structural MRI data were acquired with a 3‐Tesla Siemens TRIO scanner (Siemens, Erlangen, Germany). Data acquisition involved high‐resolution, T1‐weighted, gradient‐echo images utilizing a magnetization‐prepared, rapid‐gradient‐echo (MPRAGE) sequence. The parameters for this sequence were: TR = 1900 ms, TE = 2.99 ms, slice thickness = 1 mm, distance factor = 50%, in‐plane resolution = 1.00 × 1.00, matrix = 256 × 256, and field of view = 250 mm.

### 2.3 Data analysis

Data were converted from dicom to nii format in MRIcroGL (Rorden & Brett, 2000). All preprocessing and analyses were run in CAT12 (expert mode) version 12.8.1(r1987). Data were segmented using default settings for spatial registration including shooting registration to the CAT12 MNI152NLin2009cAsym template and voxel size thickness estimation at 0.5 mm for surfaces. Surface and thickness estimation were performed for grey matter, white matter, and cerebrospinal fluid with affine and nonlinear modulated normalized output. Following segmentation, additional surface parameters were extracted including the gyrification index based on absolute mean curvature, fractal dimension for a measure of cortical complexity, and sulcus depth based on the Euclidean distance between the central surface and the convex hull (transformed with a square root function for normal distribution). The left and right hemisphere surface data were resampled and merged to a single 32k mesh. Cortical thickness was smoothed with a 12 mm FWHM kernel and gyrification, sulcal depth, and cortical complexity were smoothed with a 25 mm FWHM kernel.

An overall weighted image quality rating (IQR) was calculated in CAT12 for each participant. The IQR is a single score that summarizes noise, bias, and image resolution for image quality before and after preprocessing. As image quality can affect surface measures (Gilmore et al., 2021), a *t*‐test was run to ensure there was no significant difference in image quality between groups (mean IQR for ASMR = 2.65; mean IQR for controls = 2.63, independent samples two‐tailed *t*‐test, *p* = .7879).

The statistical design specification and estimation were run in CAT12. A whole‐brain analysis was conducted which assessed the measures voxel‐wise rather than being parcellated into regions‐of‐interest. Separate analyses were run for the resampled and smoothed thickness, sulcus depth, gyrification, and cortical complexity images. No threshold masking was applied, and an implicit mask was applied. Contrasts of ASMR > HC and ASMR < HC were run for each of the four surface measures. The statistical maps for each measure were generated in a two‐step process. First, the results were called with a *p* value of .001 and an extent threshold of 0 in order to obtain an empirical value for the expected number of vertices per cluster (*k*), specific to each map. Second, the results were then displayed with a *p* value of .001 and the extent threshold set to the map‐specific *k* value. This process addresses multiple comparisons and reduces false‐positives. Results are reported labeled with the Desikan‐Killiany DK40 atlas.

## 3 RESULTS

**FIGURE 1:**
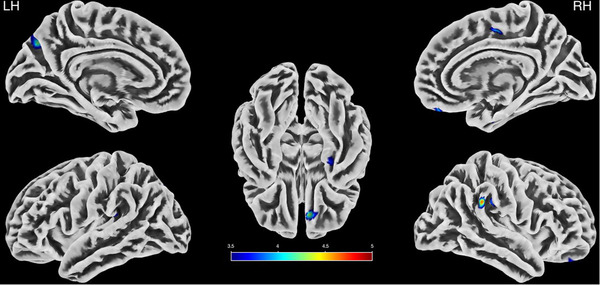
Regions of cortical thickness decreased in ASMR as compared with HC. The lateral and medial surface views of the left hemisphere (LH) and right hemisphere (RH) and the bottom surface view are displayed with the significant *t*‐values represented in the color bar.

The analysis of cortical thickness revealed several significant differences between ASMR and control participants (all *p* values < .001, with an extent threshold of *k* = 19; see Figure 1). In the left hemisphere, ASMR was associated with less cortical thickness in four regions: a 94‐voxel cluster equally distributed across the precuneus and superior parietal lobule (*T* = 4.3), a 24‐voxel cluster almost entirely within the precentral gyrus (*T* = 4.0), a 21‐voxel cluster in the supramarginal gyrus (*T* = 3.6), and a 20‐voxel cluster in the insula (*T* = 3.7). Five regions showed less thickness in the right hemisphere of individuals with ASMR. Two of these clusters were in the supramarginal gyrus (44 (*T* = 3.9) and 43 (*T* = 4.6) voxels). A 40‐voxel cluster was spread evenly between the medial and lateral orbitofrontal cortex (*T* = 4.4). A fourth region (36 voxels; *T* = 4.0) encompassed parts of the superior frontal gyrus and paracentral lobule, while a fifth region (21 voxels; *T* = 3.8) included the fusiform and entorhinal cortex. No significant differences were observed for the ASMR > HC contrast.

**FIGURE 2:**
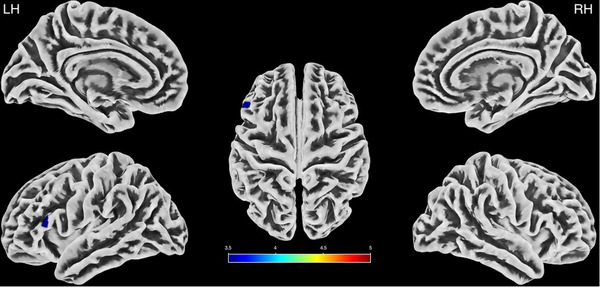
Regions of cortical complexity decreased in ASMR as compared with HC. The lateral and medial left hemisphere (LH), right hemisphere (RH), and the top surface views are displayed with the significant *t*‐values represented in the color bar.

The analysis of cortical complexity also revealed a significant difference between groups (*p* < .001 and extend threshold, *k* = 42, applied). ASMR was associated with significantly less cortical complexity in a 48‐voxel (*T* = 3.7), left‐hemisphere region comprising parts of the pars opercularis and pars triangularis (see Figure 2). No significant increases in cortical complexity were detected for the ASMR group as compared with the HC group.

ASMR participants also showed greater sulcal depth in a 50‐voxel (*T* = 3.7) region of the left inferior temporal lobe and increased gyrification in a 36‐voxel (*T* = 3.9) cluster in the right fusiform gyrus (*p* < .001). However, these results were not significant when the extent threshold calculated for these maps (*k* = 58) was applied to correct for multiple comparisons.

## 4 DISCUSSION

The current research provides the first evidence of structural differences in the brains of individuals with ASMR. ASMR was associated with reduced cortical thickness in several areas involved with phonological processing, sensorimotor functions, and attention. Two of these regions—the precuneus and precentral gyrus—also showed atypical functional connectivity in earlier research with these participants (Smith et al., 2019b). This link between cortical thickness and atypical functional connectivity is consistent with previous research with different populations [e.g., (de la Cruz et al., 2021)].

The specific brain areas showing reduced cortical thickness are also relevant to the phenomenology of ASMR. Both the left and right supramarginal gyri were thinner in individuals sensitive to ASMR stimuli. The supramarginal gyrus is involved with a number of phonological functions including the perception of pitch and rhythm (Schaal et al., 2017) and phonological working memory (Deschamps et al., 2014). The lower cortical thickness found in the ASMR participants in our study suggests that phonological information may be processed differently by this population. This supposition is consistent with the fact that auditory stimuli such as whispering and repetitive noises can elicit ASMR tingles in these individuals [e.g., (Fredborg et al., 2017)].

Reduced cortical thickness was also detected in several regions related to movement and bodily awareness. The left precuneus is associated with a number of ASMR‐relevant functions including body part localization and awareness (Felician et al., 2004), as well as perspective taking (Cavanna & Trimble, 2006, Petrini et al., 2014). Many ASMR videos involve a protagonist speaking directly to the camera, thereby immersing viewers into a body‐focused experience such as brushing one's hair. Atypical functioning in the precuneus and the adjacent superior parietal lobule may alter how an individual integrates this external information with their body's own sensations. Similarly, differences in the insula may relate to altered processing of interoceptive information (Craig, 2009). Given that this region is a cortical “hub” with dense projections to numerous regions related to sensorimotor, attentional, and emotional processes, it is possible that the differences in cortical thickness could influence several other cognitive and emotional functions (Ghaziri et al., 2017).

Our analyses also indicated that two regions of the right prefrontal cortex were thinner in individuals with ASMR. One cluster included portions of the medial and lateral orbitofrontal cortex. This cortical region is associated with numerous sensory and emotional functions (Rolls et al., 2020); neuroimaging studies have also shown that these areas are active during some forms of social cognition (Grossmann, 2013), including an analysis of social interactions similar to those found in many ASMR videos. We also detected reduced cortical thickness in a cluster including the right superior frontal gyrus, extending into the paracentral lobule. This result is noteworthy for two reasons. First, the paracentral lobule was active when these participants viewed ASMR videos in a functional neuroimaging study (Smith et al., 2019); it is possible, therefore, that the ASMR‐relevant neural activity is related to cortical thickness. Second, this brain area has also been linked with inhibitory attentional processes and to the analysis of motor urgency (Hu et al., 2016). We could therefore speculate that ASMR is related to reduced attentional inhibition. Consistent with this hypothesis, Wang et al. (2020) found that ASMR‐sensitive individuals had slower inhibitory control and set‐shifting abilities after exposure to ASMR videos.

The analyses of cortical complexity revealed that ASMR was associated with less complexity in regions that comprise Broca's area. This region is typically associated with language production; however, functional neuroimaging studies have shown that it serves some motoric functions as well, including the interpretation of others’ actions (Fadiga et al., 2006). It is therefore possible that differential sensitivity to the social movements seen in many ASMR videos is related to differences in Broca's area. Future research could address this possibility by examining cortical complexity in ASMR‐sensitive individuals who do or do not respond to this particular ASMR trigger (see Smith et al., 2020 for an examination of functional connectivity differences associated with sensitivity to different ASMR trigger types).

Although the current research provides the first investigation of structural brain differences in ASMR, it is important to acknowledge its limitations. Additional studies with larger sample sizes are necessary in order to detect further differences. Additionally, it is unclear whether the groups differed on cognitive abilities, mood, or personality variables; these factors could have weakened our ability to detect differences between the two groups of participants. Finally, this study focused on cortical brain areas. Future studies using volumetric analyses to measure subcortical structures and diffusion tensor imaging to measure white‐matter pathways would allow researchers to provide a complete depiction of the neural architecture underlying ASMR.

## CONFLICT OF INTEREST

This statement confirms that none of the authors report any conflict of interest related to the performance of this research.

### PEER REVIEW

The peer review history for this article is available at https://publons.com/publon/10.1002/brb3.2894.

## Data Availability

Data are available from the corresponding author upon reasonable request.
